# Cortical γ-oscillations implement basic language operations: Evidence from electroencephalography in anaphora during english filler-gap dependency processing

**DOI:** 10.1371/journal.pone.0333820

**Published:** 2025-10-21

**Authors:** Laurent Dekydtspotter, A. Kate Miller, Kent Meinert, Jih-ho Cha, Jae Hyun Ahn, Kyle Swanson, Yanyu Xiong

**Affiliations:** 1 Indiana University, Second Language Studies, Bloomington, Indiana, United States of America; 2 Indiana University, World Languages and Cultures, Indianapolis, Indiana, United States of America; 3 Indiana University, Speech, Language, and Hearing Sciences, Bloomington, Indiana, United States of America; 4 Purdue University, Oral English Proficiency Program, West Lafayette, Indiana, United States of America; 5 Alabama Life Research Institute, University of Alabama, Tuscaloosa, Alabama, United States of America; University of Missouri Columbia, UNITED STATES OF AMERICA

## Abstract

Hypotheses about top-down structure building propose that gamma (>30Hz) oscillations support the creation of syntax-semantics objects, contrasting with slow-rhythm entrainment of gamma oscillations tracking semantic fitness in bottom-up chunking. In bi-clausal filler-gap dependencies, like *which message regarding/about him did Frank/Diana say__ that Diana/Frank received__ unexpectedly?*, *wh*-fillers link to gap*__* sites. Although adjunct noun-phrase modifiers (Mods) like *regarding him* and noun complements (Comps) like *about him* have equal contextual fitness, in anaphoric dependencies Mods are biased toward discourse coreference and Comps toward syntactic binding enabled by *wh*-dependencies. Anaphora resolution across syntax and discourse has been tied to increased power in low- and high-gamma oscillations in retrieval and integration of referential elements. This predicts gamma-range event-related power differences (ERPDs) as anaphora processes for Comps vs. Mods interact with matrix- vs. embedded-clause antecedents at gap sites in embedded-clause *wh*-dependencies, as clause-edge gaps support Comp-enabled syntax-constrained retrieval of referential elements in binding and as thematic gaps support referential relations into the sentential interpretation. Using cluster-based nonparametric permutation tests, Mod- vs. Comp-based anaphora-linked ERPDs were examined within 30–50 Hz and 50–90 Hz in evoked activity at *say__* and *received__* in 23 right-handed English speakers to capture cell-assembly formation in the creation of referential relations for Mods vs. Comps in *wh*-filler re-representations. Gamma-power differences for Mods vs. Comps in N1 antecedent-pronoun match vs. N1 antecedent-pronoun mismatch/N2 antecedent-pronoun match arose 30–50 Hz and 50–60 Hz at *say__,* and 50–90 Hz at *received__*. Mod vs. Comp modulations of anaphora-linked gamma ERPDs at gap sites suggest that gamma oscillations implement referential relations in complex *wh*-dependencies.

## Introduction

Oscillatory activity in various rhythms of brain activity (i.e., delta (δ,.5–4 Hz), theta (θ, 4–8 Hz), alpha (α, 8–13 Hz), beta (β, 13–30 Hz), and gamma (γ, > 30 Hz; low γ 30–50 Hz, and high γ > 50 Hz) in adults) has been linked to specific cognitive functions for speech and language [1–15, inter alia]. α oscillations generally mediate the (dis)engagement of cell assemblies enabling the focus of resources [16]. β oscillations manage sentence-level information supporting prediction [8]. θ plays a crucial role in item storage and retrieval [10,12]. δ oscillations track sequential phrasal arrays [17,18]. γ has been linked to syntax [19,20] and semantics, including anaphora [21–23]. Notably, the role of γ oscillations in structuring is subject to a very lively current debate on the neurocognitive nature of syntax. Bottom-up chunking approaches to language associate syntactic structures with the tracking of sequential phrasal arrays in slow δ oscillations [3,18,24–26, inter alia], with γ oscillations tracking the fitness of ongoing contextual integration [18,25] in entrainment from processing in lower rhythms. In contrast, recent top-down structure-building hypotheses propose that γ-range activity essentially implements a basic syntax-semantics (syn-sem) operational workspace for language [6,13,24,27,28].

Presenting the most detailed oscillatory model to date, Murphy [13] proposed a representation-operation-structure-encoding (ROSE) architecture for language. In ROSE, neuronal ensembles for distinct syn-sem linguistic features for abstract lexico-grammatical expressions (that end up externalized in the sensory-motor system for sounds or for gestures) spike into local circuits’ field potentials, which essentially places expressions into a γ operational workspace. Circuital processes for generating new complex objects must involve coactivations of neuronal ensembles for lower and larger representational levels across the various subdomains of language. γ oscillations engage with θ oscillations for item storage and retrieval in conjunction with δ oscillations representing structures in multiplexed oscillatory relations [13]. Murphy’s [13] architecture echoes a cortical-hippocampal processing loop proposed for anaphora processing by Nieuwland and Martin [22] and further supported by Coopmans and Nieuwland [29]. Indeed, anaphora resolution has already been tied to increased power in low γ (around 40 Hz) for retrieval and in high γ (60–80 Hz) for integration [22].

We argue that referential processes dependent on a computational moment of recursion in complex filler-gap dependencies in anaphora resolution can address the potential role of a γ operational workspace in a processing loop architecture enabling recursion. Recursion in long-distance *wh*-filler-gap dependencies is well supported. First, in some languages, such as Afrikaans [30], German [31], as well as in child English [32], some filler expressions may be repeated at the clause edge. Second, psycholinguistic evidence across languages at the gap site is also consistent with clause-edge re-representations and filler reactivation [33–35] and with the effects of this filler re-representation on the later thematic gap site [34,36,37]. For adult first language speakers of English, Gibson and Warren [36], Fernandez et al. [34], Marinis et al. [38], and Pliatsikas and Marinis [37] also provided evidence that bi-clausal dependencies (*The manager who the secretary claimed that the new salesman had pleased will raise company salaries*) provide processing load relief at the thematic gap site relative to filler-gap dependencies of similar word length without recursion (*The manager who the secretary’s claim that the new salesman had pleased will raise company salaries*). This effect is explained if filler re-representations at the intermediate gap site refresh the filler in item memory, facilitating its maintenance over long-distance structures. Third, event-related potential (ERP) research on *wh*-movement further supports the processing load induced by filler-gap dependencies [39–45] with a sustained anterior negativity, due to storing the *wh*-filler across a dependency, and P600 effects at gap sites.

As pointed out by Kazanina and Tavano, “The endeavor of mapping linguistic computations to neural substrates requires an explicit parsing theory that outlines a detailed algorithm for how various computational subprocesses are executed in real time to yield a higher-level representation” [6, p. 123]. From a generative perspective, parsing involves the real-time deployment of grammatical knowledge, guided by representational and computational economy in cycles of processing [46–48, inter alia]. Parsing the input requires the mechanistic creation of basic linguistic objects based on feature-specific neural ensembles and their integration into structures, together with the activation of vocabulary items mapping syn-sem elements to phonological representations [49]. Building on the working neurophysiological hypothesis that cortical γ oscillations implement basic operations as in Murphy’s ROSE architecture [13], Dekydtspotter et al. [27] reported evoked activity in low and broadband γ in the implementation of anaphoric relations in first and second language French at the embedded-clause edge during the processing of bi-clausal *wh*-filler-gap dependencies. They argued that these anaphora-linked clause-edge effects support a processing loop implicating a γ-implemented operational workspace for basic operations. These operations implicate coactivations between cortical neuronal sites across levels of representations in information processing. Coactivations across such sites allow for operations such as the combining of various linguistic elements across syntax and semantics, adding features to elements in valuation, as well as the assignment of categories in labeling. This mechanistic system aligns with the basic syntactic operations required in parsing as evidenced by computational models [50–53]. A cortical γ-implemented operational workspace interacting with a general workspace for items and structures in a processing loop could support the biocomputational and psychological feasibility of Minimalist language design in parsing [50,54].

As Kazanina and Tavano note, “the development of a neurocognitive model of syntactic structure building would take as an essential starting point a model of a psychologically plausible parser. In this case, the objective is to translate into neural terms the parsing process as defined in psycholinguistics and/or computational linguistics” [6, p. 126]. Using specific characteristics of English grammar in bi-clausal filler-gap dependencies, this article examines how γ oscillations can implement referential “relationships between hierarchically organized elements” [24, p. 723] for discourse coreference and for syntactic binding as part of a processing loop allowing for multiple dependencies. Indeed, as filler-gap dependencies such as *Which message regarding/about him did Frank/Diana say __ that Diana/Frank received __ unexpectedly?* are processed, anaphora-linked event-related power differences (ERPDs) modulated by these *wh*-filler structures arise as the *wh*-filler *which message regarding him* induces discourse coreference and as the *wh*-filler *which message about him* induces structurally constrained syntactic binding in re-representation at clause-edge and thematic gap positions. While *him* can certainly be understood as referring to an individual not mentioned in the above sentence, in anaphoric relations, language allows for referential chains in which referential expressions refer to the same entity. Such referential chains can be implemented within a cortical γ**-**implemented operational workspace in basic object formation, in which, for instance, a combination of referential expressions (e.g., {Frank, him}) can be mapped to a single discourse referent x linked to an entity named Frank (e.g., {x, {Frank, him}}) in referential chain object creation for syntactic binding [27]. We argue that complex *wh*-dependencies naturally allow for the building of anaphoric relations between *him* and *Frank* across verbal modifiers (Mods) such as *regarding him* and lexically selected prepositional complements (Comps) such as *about him*. *Wh*-fillers, such as *which message regarding/about him*, are eventually associated with the embedded-clause verb. However, these Mod vs. Comp structures favor distinct referential processes in anaphora resolution: discourse coreference for propositional verbal Mods and syntactic binding for prepositional Comps. Thus, the Mod- vs. Comp-modulation of anaphoric processes refers to the downstream computational effects of lexical characteristics. Multiclause linguistic computations are typically assumed to involve recursive silent re-representations of the *wh*-filler into the embedded clause through the boundary between clauses. This recursive re-representation explains that *Which picture of himself did Frank say __ that Bill received __ unexpectedly?* may constitute a question about a picture of Frank or about a picture of Bill. In contrast, the statement *Frank said that Bill received a picture of himself* can only be a statement about a picture of *Bill,* since an anaphor like *himself* must be structurally dependent on an antecedent expression within a local binding domain. In re-representation, therefore, referential expressions enter different phases of computations.

Thus, in *which message regarding/about him did Frank say that Diana received*, an anaphoric interpretation of the pronoun *him* dependent on an antecedent *Frank* can involve a syntactic relation at the level of the grammar or a coreference relation at the level of discourse representation. At the level of the grammar, a structure such as [*Frank said Diana* [*received a message about him*]], with *Frank* outside of the default binding domain constituted by the embedded clause, allows the pronoun *him* to become structurally dependent on the matching c-commanding syntactic antecedent in syntactic binding, which involves “hierarchical syntactic relationships between words or groups of words” [24, p. 114]. This hierarchical structure allows a syntactic referential chain [him, Frank] in which the pronoun *him* is dependent on the antecedent *Frank*, as grammatical elements for the antecedent and the pronoun map together to a single discourse referent. At the level of discourse, a coreference relation instead involves mapping separate discourse referents for the pronoun and for the antecedent to the same individual in the cognitive model for some possible world [55]. In bi-clausal filler-gap dependencies, Mods such as *regarding him*, which are added to the lexically determined structure, need only be interpreted once. Hence, in *which message regarding him, him* can be immediately mapped to a discourse referent and later identified as an antecedent is encountered. In contrast, lexically specified Comps, such as *about him*, are part of the specification for the noun *message* and must, therefore, be present at every step of processing of the *wh*-filler. This computation automatically enables a grammatical dependency at any gap site after *Frank* has been encountered, in which *him* is syntactically bound to gender-matched *Frank* (see [56–60], as well as discussion below).

In sequences such as *which message about him did Frank/Diana say __ that Diana/Frank received __ unexpectedly, say* allows for prediction of an embedded-clause dependency, including *wh*-filler re-representations at the clause edge and at the embedded-clause verb. In turn, this prediction enables a hierarchical (c-command) relation between the antecedent and pronoun, as in sentences like *Frank*_*i*_ *said Diana received a message about him*_*i*_. *Wh*-filler re-representations, therefore, support the creation of referential chain objects implicating the syn-sem elements for *Frank* and *him* in syntactic binding or discourse referents for these elements in discourse coreference. The implementation of such referential chain objects requires the low-γ retrieval of relevant referential elements from item memory through synaptic input to the cortex and the high-γ formation of the referential chain objects in cortical output, so that these objects can be stored in item memory and integrated in structural memory [13]. Crucially, Mod vs. Comp structures are equally plausible and carry similar information. Hence, γ power differences arising as *wh*-fillers are re-represented enabling anaphora resolution through syntactic binding can speak to the role of the γ range in the implementation of basic operations in syntax and semantics, rather than to contextual fitness in bottom-up chunking. We first present this study’s integrative goals, addressing the relevant linguistic structures, objects, and operations in syntactic and discourse anaphora, as well as the implementation of language objects and operations by basic circuits. Based on this implementation, we next provide empirical research questions, hypotheses, and testing and analysis procedures. Following the presentation of results, the discussion addresses how oscillatory dynamics in γ can provide pathways for integrating linguistics with neuroscience to advance the understanding of the nature of language in the brain.

### Aims: Relations in language and mechanistic processes

Our first empirical research goal is to document γ-range activity in top-down structure building in the domain of anaphora enabled by *wh*-filler re-representations in intermediate and thematic gap positions. Assessing the role of γ in the implementation of basic operations in language interpretation and generation can be achieved by documenting specific, structurally dependent oscillations as basic referential chain objects are created in the implementation of anaphora resolution, as a hierarchical dependency between expressions [6,13,24,27]. A second research aim is to characterize the creation of referential objects by implementational cortical circuitry within the γ range as different types of anaphoric relations are computed in ongoing interpretation. Understanding γ-range processes more precisely can also be achieved by establishing the neurocognitive mechanisms enabling syntax-level referential objects for Comps and discourse-level referential objects for Mods. A third empirical goal is to document γ oscillatory effects across languages and constructions—with possible variations as different languages and constructions offer different orders of grammatical information in processing—to fully establish the nature of these effects.

First, the Mod vs. Comp difference constitutes a basic grammatical distinction with syn-sem effects. In Comps, such as *message about Frank*, the preposition *about* is lexically required as a feature of the noun *message* (see *message* *of/√*about*). Semantically, the structure *message about* constitutes a function linking entities to related communications. The noun licensing a complement is viewed as relational. Such structures minimally involve a basic combination (e.g., {*message*, *about Frank*}) labelled by a head (e.g., the underlined nominal element in {*message*, {*message*, *about Frank*}}), with an interpretation as function application which blends the two elements [57]. Grammatical extraction is possible out of Comps, as in *Who did Frank say that Diana received a message about?* even if long-distance dependencies are costly and stranded prepositions are criticized as improper speech by purists. In Mods, such as *message regarding Frank*, the expression *regarding Frank* offers additional rather than relational information. Chomsky [57] argued that Mods involve a distinct operation: pair-Merge. Pair-Merge conjoins the two expressions into the ordered pair <*message*, *regarding Frank* > , which can be defined as {{*message*}, {*message*, *regarding Frank*}} [61], so that the Mod expression characterizes the relevant communications both as messages and as relating to Frank. As a result of this structure, extraction out of a modifier is ungrammatical: *Who did Frank say that Diana received a message regarding? Hence, if *message about Frank* and *message regarding Frank* provide the same overall information, they do so with different grammatical processes: in a lexically encoded relational fashion for Comps and as additional post-lexical information for Mods.

Second, pronouns, as grammatical elements valued contextually in syntax or in discourse, also have top-down constraints on their interpretation. Pronouns like *him* must be free within a local binding domain that contains them, typically a clause containing a subject, unlike anaphors like *himself* that must be bound (i.e., syntactically dependent on an antecedent) within this domain [62]. Thus, in *John said that Bill saw him/himself*, the pronoun *him* cannot refer to *Bill,* whereas the anaphor *himself* must refer to *Bill.* Indeed, *Bill* resides within the local binding domain for *him*/*himself,* the embedded clause. The pronoun *him*—but not the anaphor *himself*—can, however, refer to *John* in discourse or be bound to *John* in syntax, since *John* resides outside the embedded-clause binding domain. A nominal can constitute a binding domain, but only if it has a nominal subject. Hence, in *John repeated Bill’s story about himself,* the anaphor *himself* must be syntactically dependent on the nominal subject *Bill*. In contrast, a pronoun as in *John repeated Bill’s story about him* is valued outside the nominal. *John repeated a story about him* is also possible with *John* as binder for the pronoun *him*. However, in this case, the story must originate with someone other than John. Chomsky [62] argued that in such cases, a silent nominal subject allows a nominal binding domain of last resort, when a pronoun inside a Comp has an antecedent within the clause. End-of-sentence processing evidence also aligns with this claim [63].

Therefore, the basic Mod vs. Comp difference interacts with referential processes in long-distance dependencies in strings like *which message regarding/about him did Frank say that Diana received unexpectedly,* allowing the processing to be observed experimentally [27,64]. Long-distance *wh*-dependencies have been argued to involve silent *wh*-filler re-representations, described as silent copies (or as movement traces, indicated as *t*) in recursive steps through the embedded-clause edge into the thematic position as in (1a, b) [62,65,66].

(1a) [[which <message, regarding him>] [did [Frank say [*t* [that Diana received *t* unexpectedly]]]]]
(1b) [[which {message, about him}] [did [Frank say [*t* [that Diana received *t* unexpectedly]]]]]

Psycholinguistic evidence (priming [35]; pupillometry [34]; self-paced reading [36–38]) has been found to be consistent with the computation of *wh*-filler re-representations in intermediate clause-edge and thematic gap positions, with clause-edge re-representations facilitating thematic integration relative to constructions without such re-representation. The Mod vs. Comp distinction in *wh*-dependencies influences referential relations, with Mods as conjoined expressions residing in a separate dimension from the computation based on lexical selection [57]. Mods may, therefore, be interpreted only once during the dependency, whereas lexically required Comps are obligatorily linked to every step of computations implicating the head noun (see [56–60], for extensive discussion). For instance, the string *which message regarding Frank did the idiot say that Diana received unexpectedly* allows the expression *the idiot* to anaphorically refer to *Frank*. However, in the string *which message about Frank did the idiot say that Diana received unexpectedly,* the expression *th*e *idiot* must refer to someone other than Frank, similarly to the fact that *the idiot* cannot refer to Frank in *The idiot said that Diana received a message about Frank.*

Likewise for pronouns, even when the information conveyed is similar across Mod and Comp structures, as in the string *which message regarding/about him did Frank say that Diana received unexpectedly*, distinct computations are expected, given that Mods, unlike Comps, need only be interpreted once. In *regarding him*, the pronoun can be assigned a discourse referent in the sentence-initial position, with the referent later identified as Frank. In *about him*, however, the evaluation of *him* requires an embedded-clause binding domain with the *wh*-filler re-represented at the clause edge (and at the thematic gap inside it). This enables a structural referential binding relation in which the grammatical elements for *him* and *Frank* form a referential chain object. As noted earlier, an embedded-clause binding domain also allows for a discourse-level coreference relation for the pronoun in *about him*. Thus, as a *wh*-filler is re-represented as in the string *which message about him did Frank say that Diana received unexpectedly,* an embedded-clause structural configuration in slow rhythms [3,13] induces a referential chain object computation involving the antecedent *Frank* and pronoun *him* implemented in the cortical γ-implemented operational workspace. Hence, in a processing loop involved in the implementation of relations in syntax as well as in discourse for “hierarchically organized elements” [24, p. 723] the *wh*-movement configuration with a Comp structure licenses the creation of a referential chain object for the elements *Frank* and *him,* implemented by γ oscillations at specific computational moments.

Using French, which grammatically marks verbal Mods (*le concernant,* ‘concerning him’) vs. prepositional Comps (*à propos de lui* ‘about him’), Dekydtspotter et al. [27] argued that such structures provide a way to examine a γ-implemented syn-sem operational workspace functioning across first and second language backgrounds. Hence, *wh*-fillers qualified with Mods vs. Comps predict specific ERPD patterns reflecting syntax-based anaphora for Comps vs. discourse-based anaphora for Mods at different computational moments. In addition, since β rhythm activity tracks the maintenance of sentential information enabling prediction, Dekydtspotter et al. [64] also reported β-range ERPDs in induced power that signaled differences in processing load for Mod (*le concernant*, ‘regarding him’) vs. Comp (*à propos de lui*, ‘about him’) structures in anaphora in French, in both first- and second-language speakers. β-range sentential processing load effects were associated with the extraction of bridge verb information and confirmation at the subordinator of a tensed embedded clause, with evidence of greater processing load among second-language speakers. These effects were timed with clause-edge computations for syntactic binding vs. coreference in anaphora resolution. They revealed greater power for lexically encoded Comps (vs. Mods) in matrix-clause antecedent match in anaphora resolution, and greater power for Mods (vs. Comps) in matrix-clause antecedent mismatch as anaphora resolution is delayed. These effects are consistent with binding vs. coreference processes of anaphora resolution for Comps and Mods and with the need to maintain an unidentified discourse referent across the clause edge in coreference vs. binding as anaphora resolution is delayed.

Addressing the role of the γ rhythm in the computation of such dependencies at the clause edge, Dekydtspotter et al. [27] examined γ-range ERPDs in evoked power at the clause edge as cell assemblies formed in referential processing. γ power differences matching those asymmetries reported in β occurred at the subordinator in low γ, followed by broadband-γ effects, consistent with the retrieval of information in low γ and integration in high/broadband γ. Ultimately, however, establishing γ oscillations as a cortical operational workspace executing syntactic vs. discourse referential relations in anaphora requires finding effects across languages and constructions. In the current study, we examine Mod vs. Comp differences in English, using bi-clausal interrogative structures with two important differences from the French structures tested by Dekydtspotter et al. [27]: First, the morphosyntax of the pronoun remains the same across Mod *regarding him* and Comp *about him*, unlike in French. Second, lexico-grammatical elements provide a different timing of information as *did* marks interrogative and past-tense features and unambiguously requires a main verb, which limits the need for reanalysis, relative to French, in which past tense auxiliary *avoir* ‘have’ in *a dit* ‘said’ can also have a present-tense main verb alternative value.

A fuller integration of Minimalist approaches to linguistics with neuroscience minimally assumes that features, basic elements, operations, as well as phrasal representations are implemented by simple neuronal assemblies [66] and that a neurolinguistic model for language must account for parsing [6]. The biocomputational basis of Minimalist approaches is well established in parsing research [50,67]. In left-corner parsing, sentence processing involves bottom-up and top-down processes implemented in any order consistent with incremental processing [48,54]. In incremental parsing of a *wh-*filler-gap dependency, a *wh-*determiner phrase must be structured within a D(eterminer) > *n*(ominal)> root √N(oun) concept category hierarchy for nominal expressions (Table 1) and must be integrated within a C(omplemetizer) > T(ense) > *v*(erbal)> root √V(erb) concept hierarchy for clauses (Table 2). Crucially, a binary γ-implemented cortical operational workspace for basic operations (e.g., Merge, together with labeling and feature valuation) interacting with a general resource workspace enables left-corner parsing options [68], as shown in Tables 1 and 2. In incremental processing of a *wh*-filler within the γ-implemented binary cortical operational workspace, neurocognitive processes implementing basic operations involve the coactivation of cortical sites across distinct levels of representations in information processing. Hence, for instance, a *wh*-determiner element for *which* (i.e., D: *which*) can be combined with a precomputed nominal-category-level combination (i.e., *n*: Number* + *√N[0])—including a bare nominal-concept category √N, with an empty feature space [0] as a placeholder—into a set {D: *which*, *n*: Number* + *√N[0]} and labeled into a new partially specified combination {*D: *which**, {D: *which*, *n*: Number* + *√N[0]}} for externalization and interpretation [69]. In further processing, a second phase of computations can then enable the identification of root N-level contents with the conceptual root features for *message* added in valuation.

**Table 1:** Parsing of a *wh*-DP filler item within the D(eterminer) > *n*(ominal)> root √N(oun) category hierarchy in recursive deployment of γ operational workspace.

Buffer for activated lexico-grammatical items and combined expressions:	D: *which*, *n*: Number, √N[0]	D: *which*, *n + *√N[0]	{D, {D: *which*, *n + *√N[0]}} √N[0], Message
Binary γ workspace loads	[*n*: Number, √N[0]]	[D: *which*, *n + *√N[0]]	[√N[0], Message]
Merge	{*n*: Number, √N[0]}	{D: *which*, *n + *√N[0]}	{√N[0], Message}
Combined object creation in labeling and valuation	{*n*, {*n*: Number, √N[0]}}	{D, {D: *which*, *n + *√N[0]}}	{D, {D: *which*, *n + *√Message]}}
Phrasal representations in structural memory	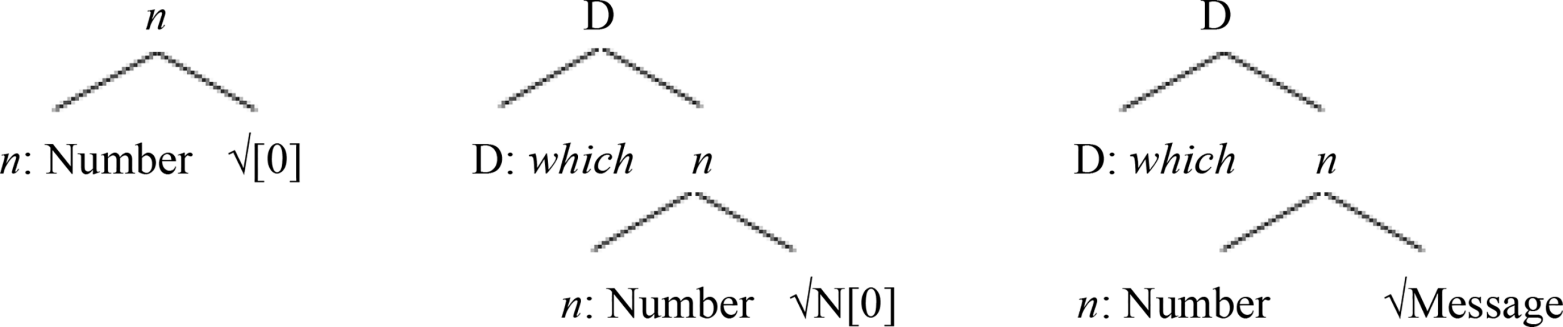		

*Note*. [a, b] represents elements loaded in an operational workspace. a + b represents a combined structure normalized to an element. √ represents a semantic root. a represents a category assignment. The integration of the noun category involves the valuation of a placeholder for nominal concepts, implicating the union of the empty set for the placeholder with the feature set for the nominal concept.

**Table 2:** Parsing of *wh*-DP dependencies within the C(omplemetizer) > T(ense) > *v*(erbal)> root √Verb hierarchy for clauses in the γ operational workspace.

Steps	(1) initial *wh*-DP integration	clause-edge (2)...and embedded verb (3) *wh*-DP re-integrations	
Binary γ workspace loads	[C[wh*, Q]+T[0], *wh*-DP]	[C[wh*]+T[0], *wh*-DP]	[√Verb[*u*D], *wh*-DP]
Merge	{C[wh*, Q]+T[0], *wh*-DP}	{C[wh*]+T[0], *wh*-DP}	{*wh*-DP, √Verb[*u*D]}
Label	{C[Q], {C[Q]+T[0], *wh*-DP}}	{C, {C[wh*]+T[0], *wh-*DP}}	{√Verb, {*wh*-DP, √Verb[*u*D]}}
Phrasal representations in structural memory	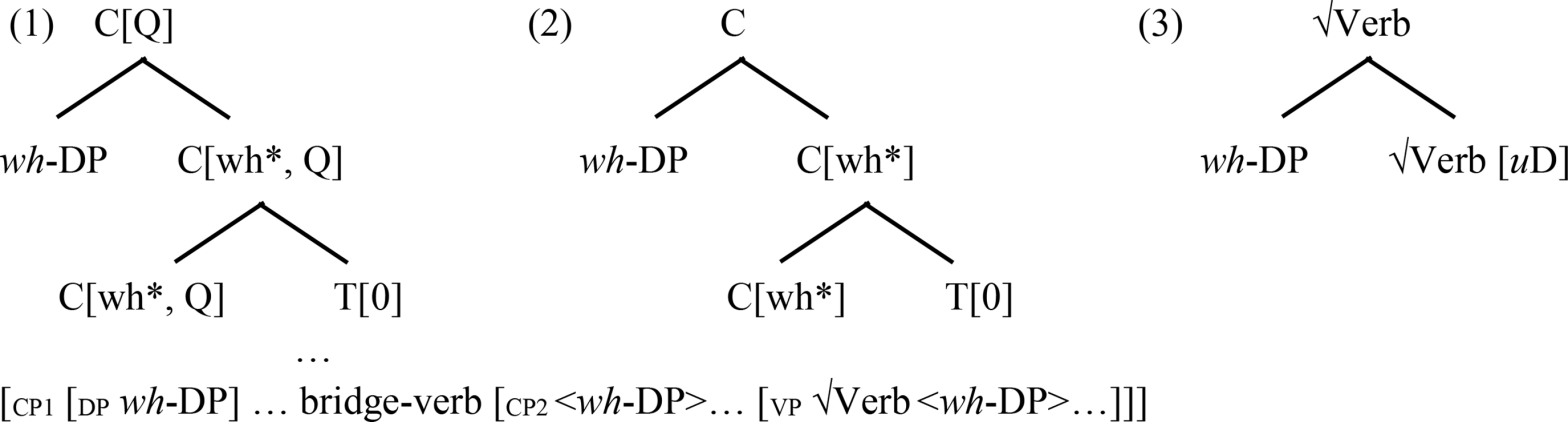		

*Note*. [a, b] represents elements loaded in an operational workspace. a + b represents a combined structure normalized to an element. √ represents a semantic root. a represents a category assignment. The integration of each phase involving the valuation of a placeholder as in Table 1 is reflected in the phrasal structure.

Computational efficiency is expected to guide parsing. The derivation in Table 1 starts by merging an *n*: Number category element, containing an unspecified Number value, with an empty root nominal-concept placeholder √N[0] for the bare category (column 2). The D: *which* element is then merged as the stored combination is retrieved into the workspace and is normalized to a basic element (column 3). The next computational step involves the computation of the root N-level representation in additive valuation (column 4). An alternative derivation in top-down D > *n* > √N processing would start by merging the D-category with a noun-category placeholder *n*[0]. Its valuation requires merging a *n*: Number element with a nominal-concept √N[0] placeholder. Its valuation then requires additional cycles of operations as the root-level information is added. Under Minimal Yield, “the fewest possible new items accessible to further operations” should be constructed [70, p. 19]. The derivation in Table 1 diminishes the number of combinations by selecting as much information as possible.

The *wh*-filler element encoded in item memory can be recursively reinstated to the γ-implemented cortical operational workspace, to be merged with various sentential category objects in additional phases of computation as shown in Table 2 columns 2–4. Hence, in classical feature-based approaches to *wh*-movement, *wh*-fillers and gaps reflect strong wh*-features triggering movement to the interrogative clause-edge category. Additionally, the extended projection principle (EPP) requires strong uninterpretable category features [*u*D*] for movement between argument positions [71]. Therefore, in the top-down parsing of *wh*-filler-gap dependencies, the *wh*-filler phrase (i.e., *wh*-DP) is re-loaded into the γ-implemented cortical operational workspace together with a normalized interrogative category object C[Q, wh*]*+*T[0] to be merged in set formation and receive a label in a bare phrase structure design (see Table 2, column 2). This clause-edge category object enables the *wh*-filler to restrict the question operator (Q) [72–74]. It also contains a T-category placeholder T[0] to be valued in the structural integration of the next phase of computations with the hierarchy. The next phase is, therefore, triggered by the need for T-category content. Under the EPP, a tense-marked category requires that a determiner phrase be merged with a tense category object, T[*u*D*]+*v*[0]. In incremental processing, the *wh*-DP element is therefore reloaded from item working memory into the binary operational workspace together with a normalized tense category object in further Merge {*wh*-DP, T[*u*D*]+*v*[0]} inducing the active filler strategy [75,76]. Upon labeling, the new T-category phrasal object {T, {*wh*-DP, T[*u*D*]+*v*[0]}} normalized to a basic element can provide the value for the tense category placeholder T[0]. A verbal category placeholder *v*[0] to be identified allows for next updates. During the next phases of computations, the *v* category for verbal features with the verbal root concept information allows for the thematic integration of the *wh*-filler: { √Verb, {*wh*-DP, √Verb[*u*D]}}, completing the process (column 4).

Table 2 illustrates a classical feature-based account of movement that echoes complementizer agreement in *wh*-movement as in Irish [77], together with the expression of clause-edge copies [30–32]. It reflects an automatic Merge-Label system in coactivation between sites across levels of representations. However, since transfer to the interfaces requires a label, movement dependencies have been argued to reflect the timing of the labeling process [78,79]. A labeling-based *wh*-dependency computed in phases [_CP_ *wh*-DP... {*wh*-DP, C + I[0]}] in left-corner parsing would reflect a Minimal Search-induced labeling process within the loop architecture [50,80].

Basic processes can also allow the establishment of referential chain relations in anaphora resolution during syntactic computations [27]. The position that anaphoric relations involve Merge-like processes is well established in Minimalist grammar. Kayne [81] proposed that referential relations as in *John said that Mary saw him* implicate a ‘doubling constituent’ [*John him*] together with the movement of *John* as in [John said that Mary saw [<*John*> *him*]] leaving a silent copy <*John* > . Fong and Ginsburg [52] implemented Kayne’s approach in proposing a Minimalist computational parsing mechanism. However, cases such as *The dog John petted bit him* would require a complex parallel/sideways movement [82], which has been criticized as biologically problematic [83]. Furthermore, such movement is ruled out by a dual-workspace design [84]. The ‘doubling constituent’ [*John him*] approach does not match constraints on movement computations together with the involvement of pragmatics in coreference relations but could work for anaphors like *himself* [85]. A dual-workspace design of the type considered in this paper between general working memory for vocabulary items and for structures and a cortical γ operational workspace for operations can capture aspects of Kayne’s intuitions of a Merge-like operation in referential relations for pronouns within a more traditional framework. These referential relations involve similar processes in the creation of referential chain objects: first combining the syn-sem elements *Frank* and *him* as in {*Frank*, *him*} and mapping this combination of referential grammatical elements to a single discourse referent x for an entity named *Frank* in the mental model. This mapping creates a referential chain object {x, {*Frank*, *him*}} for storage in working memory and for integration of an anaphoric relation into the ongoing sentential representation. Again, pronouns can always receive a discourse referent. However, a grammatical binding process in which multiple elements receive a discourse referent in one fell swoop eliminates the need for multiple discourse referents in anaphora, as argued by Reuland [86] and experimentally tested by Koornneef and Reuland [87].

In a string such as w*hich message regarding her did Frank say that Diana received unexpectedly,* the Mod *regarding her* need only be part of the interpretive cycle for the *wh*-filler noun phrase once. A variable discourse referent must be assigned early on to the pronoun (i.e., y: *her*), to be later identified with a referent for an antecedent (i.e., x: *Diana*) in discourse anaphora resolution. In cortical processing, the identification (x = y) of the discourse referents for *Diana* and *her* in coreference can be achieved by mapping a combination of discourse referents {x: *Diana*, y: *her*} to an entity (d) in a cognitive model for a possible world with someone named Diana [55]. This mapping creates a discourse representation object {d, {x: *Diana*, y: *her*}} for integration into the ongoing discourse representation structure. In the string *which message about her did Frank say that Diana received unexpectedly* with a Comp structure *about her* reliant on an embedded-clause domain, a nominal syntactic category antecedent placeholder can be anticipated in advance of encountering the expression *Diana* in the anticipation of a referential chain object for binding: {*n*: Animate: Gender: Feminine, *her*}. The creation of syntactic and discourse referential chain objects, to be stored in memory and integrated into the ongoing development of grammatical and discourse structures, predicts certain patterns of effects in implementational circuitry as a *wh*-dependency allows grammatical or discourse referential elements to be returned in low γ to the γ-implemented operational workspace, and then as syntax-based or discourse-based referential chain objects are created in high γ for integration into the ongoing sentential interpretation.

Crucially, the processes defining basic operations in terms of the nature of the manipulated elements involve information-preserving (Markovian) processing. Hence, processing for syntax seems compatible with computational Bayesian inference approaches to information processing by cortical circuitry. George and Hawkins discussed a generative hierarchical neural Markovian model of information processing in which “higher levels of the hierarchy represent larger amounts of space and longer durations of time” [88, p. 2]. The hierarchical γ-band processing of the type needed for basic language operations might therefore be characterizable in terms of coincident high probabilities and sequences of coincident high probabilities across coactivated processing nodes for syntactic and discourse-semantic elements, combinations, and objects, enabling information conservation in processing. The characterization of coactivation as probability patterns between nodes across the hierarchy does not negate specialized domains and feature-specific circuits, let alone internally driven computations. George and Hawkins [88], therefore, offer a potential account that highlights the fact that symbolic processing within a network may involve activations of random subsets of the circuits encoding features across domains. The elementary processes linked to the creation of linguistic objects of various natures in high γ suggest that implementational circuits for a hierarchical workspace can account for various operations as a reflex of the representations involved.

### Hypotheses, research questions, and predictions

We examined critical items like (2a-d), manipulating the contents of the *wh*-filler, with either a Mod (2a, c) or Comp (2b, d) qualification of the noun. The pronoun inside the *wh*-filler matched the gender of an antecedent either in the matrix clause (N1 antecedent-pronoun match; 2a, b) or in the embedded clause (N1 antecedent-pronoun mismatch/N2 antecedent-pronoun match; 2c, d).

(2a) *Which message regarding him did Frank say that Diana received unexpectedly?*
(2b) *Which message about him did Frank say that Diana received unexpectedly?*
(2c) *Which message regarding him did Diana say that Frank received unexpectedly?*
(2d) *Which message about him did Diana say that Frank received unexpectedly?*

In top-down re-representation of *wh*-fillers in the dependencies in (2a-d), the main verb *sa*y, as a main verb is expected after the helping verb *did,* triggers a clause-edge category linking the *wh*-filler to the embedded clause. The embedded verb provides the eventual object with the thematic role of the *wh*-filler, indicating that a message under questioning was received. The Mod structure *regarding him* in (2a, c) constitutes a binding domain, enabling a variable discourse referent to be immediately assigned to *him*, and identified as Frank in coreference. In matrix-clause anaphora computations for Comps (2b, d), a referential chain object can only be created in syntactic binding as a structural binding domain is computed, upon the integration of the *wh*-filler with the subordinate clause. With a matching matrix-clause antecedent, Comp-enabled anaphora depends on an embedded-clause binding domain, with anaphora through binding (in addition to coreference) predicting additional costs. With a mismatching matrix-clause antecedent, an unresolved variable discourse referent in Mods (2c) must be actively maintained into the embedded clause, whereas a binding chain with an anticipated structural binder {*n:* Gender: [-Feminine], *him*} avoids these costs in Comps (2d).

Table 3 provides a timing overview of anaphoric processes for Mods vs. Comps in N1 antecedent-pronoun match vs. N1 antecedent-pronoun mismatch/N2 antecedent-pronoun match involved in the formation of the referential chain objects constructed in an operational workspace, along the lines considered in Adger [84], all implemented by cortical γ oscillations. Thus, in processing strings such as w*hich message regarding/about him did Frank say that Diana received unexpectedly,* the construction of the referential chain object {x, {*Frank*, *him*}} in binding is enabled by the Comp *about him* (2b), as per line 3 in Table 3. The process of constructing the referential chain object {x, {*Frank*, *him*}} should induce greater power, as the elements for *Frank*, *him* are retrieved from working memory into the γ operational workspace and as a discourse referent is assigned to the combination {*Frank*, *him*}. These effects are expected as an embedded-clause domain enabling syntactic binding is computed in filler-gap dependencies. The discourse object {f, {x: *Frank*, y: *him*}}, *regarding him* (2a), as per line 2 in Table 3, is enabled upon processing *Frank*, and therefore need only be refreshed as the *wh*-filler is re-represented. A pre-established discourse referential chain object should therefore implicate less power within the specific window for the structural computation. In contrast, in late-matching antecedent strings such as w*hich message regarding/about him did Diana say that Frank received unexpectedly,* the processes for *regarding him* (2c), as per line 4 in Table 3, should induce greater power for the active maintenance of the unidentified discourse referent y for *him* into the embedded clause as well as for multiple referents mapping to the individual f, for someone named Frank, in the model’s entities. All of this creates the discourse referential chain object {f, {x: *Frank*, y: *him*}}. As shown in line 5 in Table 3, for (2d), Comps enable a partial referential chain object for syntactic binding {x: ¬ Feminine(x), {*n*: Animate: Gender: -Feminine, *him*}} pre-established into the embedded clause. This avoids mapping a discourse referent for the pronoun. This syntactic referential object can be later updated as {x, {*Frank*, *him*}} as a nominal binding domain is computed. As argued by Reuland [86] and by Koornneef and Reuland [87], avoiding discourse referents eliminates referential processing costs at the discourse level. This necessary syn-sem computation of a sentential structure requires fewer resources within the embedded clause than multiple referents in coreference.

**Table 3:** Timing of referential chain objects construction in syntactic binding and discourse coreference during the filler-gap dependency enabling a binding domain for the embedded clause.

structures	antecedent-pronoun match	pre-gap objects	low-γ cyclic retrieval into an operational workspace	high-γ cyclic creation of objects within the workspace
(2a) regarding him	N1	{f, {x: *Frank*, y: *him*}}		
(2b) about him	N1		[*Frank*, *him*]	{x, {*Frank*, *him*}}
(2c) regarding him	N2	y: *him*	[y: *him…*]	{f, {x: *Frank*, y: *him*}}
(2d) about him	N2		[n: [-Fem], *him*]	{x: ¬ Fem(x), {n: [-Fem], *him*}}

*Note*: [a, b, …] represents elements retrieved into the operational workspace. {a, b} represents the combination of elements into a new object. In the feature structure Gender: 0/Feminine, masculine is the absence of Feminine (i.e., “not feminine”, [-Fem]). The partial nominal structure n: [-Fem] represents an anticipated structural binder for Comps in syntactic binding resolved upon completion of the processing.

Mod- vs. Comp-modulated anaphora-linked ERPD patterns in N1 antecedent-pronoun match vs. N1 antecedent-pronoun mismatch/N2 antecedent-pronoun match, specifically timed as a binding domain is established in embedded-clause *wh*-dependency processing, should reflect the availability of syntactic binding in Comps vs. Mods. The availability of binding in (2b) vs. discourse coreference in (2a) in N1 antecedent-pronoun match, along with the cost of maintaining an unidentified referent in coreference in N1 antecedent-pronoun mismatch/N2 antecedent-pronoun match (2c vs. 2d), predict specific Mod vs. Comp ERPD patterns [27]. As Coopmans and Nieuwland [29] and Nieuwland and Martin [22] demonstrated, low-γ oscillations can be linked to the retrieval of referential elements from working memory and high-γ oscillations to semantic unification. In terms of the timing of these ERPD effects, the retrieval of referential elements from working memory and their combination should take place as soon as a *wh*-filler containing a pronoun is linked to the embedded-clause edge in re-representation. Mod- vs. Comp-modulated anaphora-linked ERPD effects in γ should first arise as an embedded-clause binding domain induces the low-γ retrieval of discourse- vs. syntax-level referential elements and their integration into new objects in high-γ computations. Hence, low-γ ERPDs due to retrieval should be followed by high-γ effects as elements are combined and as these combinations receive referential values in anaphoric interpretation enabling the consolidation of a sentential interpretation when the *wh*-filler is thematically integrated.

Therefore, turning to the role of γ oscillations, RQ1 asks: Will Mod- vs. Comp-modulated anaphora-linked ERPDs arise as the bridge verb requires an intermediate gap position, consistent with the low-γ reinstatement of referential elements to a γ-implemented syn-sem operational workspace? RQ2 asks: Will Mod vs. Comp structure-dependent anaphora-linked ERPDs then arise as the *wh*-filler is integrated within the thematic domain in high γ as referential chain objects are computed to terminate the interpretation of the structure? In response to these research questions, we expect that, due to syntactic binding allowed by Comps vs. discourse coreference processes in Mods, anaphora-linked Mod vs. Comp ERPDs should arise first in low γ as referential elements are reinstated from working memory and then in high γ as referential chain objects are computed and an anaphoric relation resolved enabling the finalization of the on-going interpretation of the sentential structure upon the thematic integration of the *wh*-fillers.

Crucially, only in specific top-down language structuring [6,13,28] should Mod- vs. Comp-modulated anaphora-linked γ ERPDs arise without being accompanied by any observable differences in the synchronous processing of phrasal arrays in Mod vs. Comp conditions. Only in specific top-down language structuring will Mod- vs. Comp-modulated anaphora-linked γ ERPDs reflect the timing of silently computed gaps in re-representation. Only in specific top-down language structuring will Mod- vs. Comp-modulated anaphora-linked γ ERPDs reflect the retrieval of referential elements in low γ as well as the computations of referential chain combinations in high γ—everything else being equal in terms of the semantic fitness of Mod vs. Comp structures. This is because in bottom-up approaches to language processing [3,24], in which higher order processes in γ oscillations respond to the tracking of phrasal arrays [18,25], such effects are not expected and would be purely coincidental. Computations that do not involve the re-representation of *wh*-fillers should also not result in such a timing of effects [89].

## Materials and methods

This research was approved by the Indiana University Institutional Review Board. At the start of the experimental session, participants read the study’s Statement of Informed Consent. They were asked whether they had any questions and whether they consented to participate in the study. Participants provided verbal consent to the researcher, to further protect participant confidentiality and anonymity, and were reminded that they could withdraw at any point. The stimuli consisted of 240 trials: 120 experimental items and 120 distractor items. The 120 experimental items were 30 quadruples as in (2a-d). 50% of trials involved masculine referents/pronouns and 50% of trials involved feminine referents/pronouns. 120 distractor items involved complex interrogative structures and permutations like the target items, counterbalanced so that no grouping stood out. The stimuli were presented via E-Prime [90], appearing in four blocks presented in random order and with randomization within each block; crucially, no two items from a set ever appeared in the same block. Thus, participants saw all variations of each item. This will allow for future testing on the same stimuli of populations with a more limited vocabulary range, i.e., second language speakers. In this task, respondents read sentences presented one word at a time at a predetermined pace. We used a 36-point Consolas font and normal orthographic conventions. Participants sat in a chair facing a computer monitor about four feet away. A fixation cross appeared at the center of the screen before each item, lasting 700ms. The total presentation time for each word was 551ms (300ms word presentation and 251ms interstimulus interval to the next word), except for the last word (300ms word presentation only). The total presentation time for each experimental item was 5811ms. The presentation of the bridge verb *say* started at 3307ms and the presentation of the embedded clause verb (e.g., *received*) started at 4960ms.

Respondents were trained to read questions like the stimuli and then complete true-false comprehension checks, which were presented in their entirety for a maximum of 3500ms. These comprehension checks were of several types: Some examined a pronoun’s anaphoric interpretation, while others queried other aspects of the sentences. Participants were asked to quickly respond to the statements by pressing the left arrow key for ‘True’ and the right arrow key for ‘False.’ There was a training session of six items, which could be repeated before moving on to the experiment. In the training, all items were followed by a comprehension check; in the task, only two thirds were. This rate maintained participant attention without being overly taxing. Naturally, a set of questions like our stimuli seems plausible in only a limited set of situations. Thus, respondents were introduced to a context involving two friends who were devoted followers of a television series. One of the friends, however, had missed some episodes and asked the other some questions to catch up.

### Participants and testing procedures

A total of 27 participants with English as their first language were recruited for this study. One participant aborted the study, leaving the total participant count at 26 (14 F, 12 M; 23 RH, 3 LH). We report results from 23 right-handed participants (13 F, 10 M). These participants were undergraduate students with no history of dyslexia. Accuracy rates of 76% on factual comprehension checks show the task to be challenging. On comprehension checks related to anaphoric interpretation, respondents interpreted the pronoun as referring to the gender-matched noun phrase 75% of the time. However, comprehension check accuracy was not used as a filter for analysis: Real-time brain processing as participants compute a bi-clausal filler-gap dependency is expected to be independent of their behavior on comprehension checks following individual sentences.

### EEG preprocessing and data extraction

**Fig 1:**
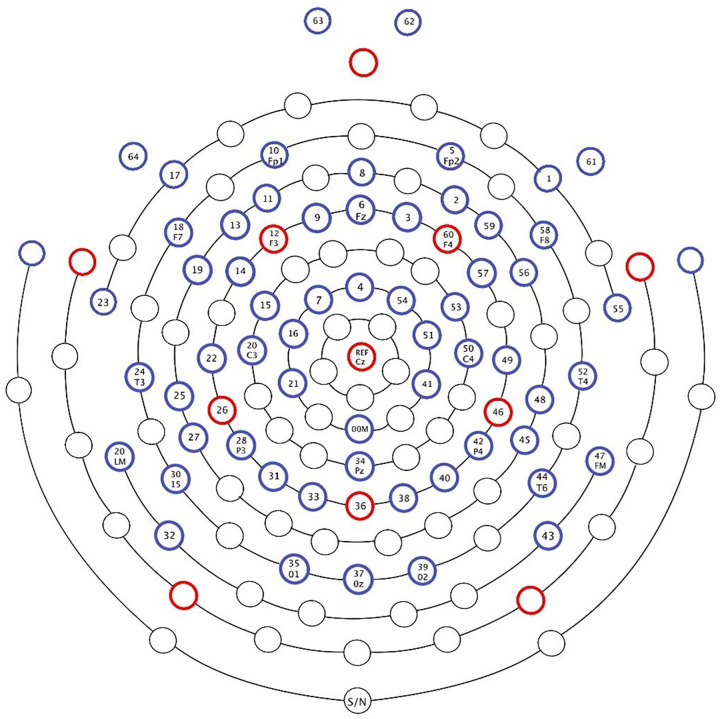
The 64-electrode EGI system.

EEG was recorded at a 1000 Hz sampling rate via a 64-electrode EGI system (Electrical Geodesics Inc., Eugene, OR; as displayed in Fig 1) referenced to Cz (vertex) online. The signal was collected using a Net Amps 300 amplifier with a gain of 5000 and acquisition software Netstation (version 4.5.4). Impedances were verified to be below 50 kΩ before each of the four blocks in the task. All preprocessing and data cleaning procedures were performed using the EEGLAB toolbox based on MATLAB (version 9.5) [91]. An 8ms latency shift due to the amplifier was corrected before preprocessing. Line noise was removed using the CleanLine plugin for EEGLAB [92].

The continuous data were then divided into 3.60-second epochs starting with *did* and running to the end of the sentence. Following segmentation, we visually inspected each epoch for bad channels and, if a channel was bad in more than 10% of epochs, we removed the whole channel. It has been demonstrated that muscle activity can create noise in high-frequency EEG measures, and γ-band results should thus be reported and interpreted with caution [93]. Hipp and Siegel [94] showed that removing such artifacts from the EEG recording through rejecting data sections affected by artifactual signals or Independent Component Analysis (ICA) can allow for more confident analysis of high-frequency EEG. Therefore, we visually inspected each epoch and systematically removed any epoch including unexpected EMG activity (i.e., furrowing of the brow, face and neck movements, but not blinks). We used the runica algorithm for ICA to extract and then manually check the 32 most impactful components generated by principal component analysis (PCA) so that we could effectively remove the remaining ocular and cardiac activity along with any remaining artifacts. An average of 89% of trials were retained across subjects (*SD* = 3.120), with an average rejection number per subject of 19.01. The average number of trials retained was similar across conditions, (2a), *M* = 27.00, *SD* = 3.37; (2b), *M* = 26.61, *SD* = 3.60; (2c), *M* = 25.83, *SD* = 3.08; (2d), *M* = 27.26, *SD* = 2.26. The data were average referenced and missing channels were interpolated for the time-frequency analysis.

### Time-frequency analysis

The preprocessed EEG data were loaded into the FieldTrip toolbox [95] as four datasets for the four structural conditions (2a - d). The time-frequency analysis was run in the time window of 3600ms from the onset of the word *did* to the end of the sentence to achieve stable frequency decomposition with a long baseline before the first analysis at the bridge verb. One might wonder if coreference computations could be entertained at the matrix subject in N1 antecedent-pronoun match conditions (2a, b) and at the embedded subject in N1 antecedent-pronoun mismatch/N2 antecedent-pronoun match conditions (2c, d). Crucially, we examined Mod vs. Comp comparisons in early vs. later antecedent-pronoun match conditions [(2a - 2b) – (2c - 2d)]. Mod- vs. Comp-modulated ERPDs in N1 vs. N2 antecedent-pronoun match are completely dependent on structural binding domain constraints on referential chain object creation. Across binding and coreference referential relations, the interpretation of pronouns in Comps depends on *wh*-filler integration within an embedded clause defining a default binding domain. In contrast, pronouns inside a clausal Mod structure defining the binding domain allow immediate coreference. This makes highly specific predictions about the timing of the processes that implement the computation of referential chain objects resulting in Mod- vs. Comp-modulated anaphora-linked ERPDs between N1 antecedent-pronoun match and N1 antecedent-pronoun mismatch/N2 antecedent-pronoun match. N1 antecedent-pronoun match vs. N1 antecedent-pronoun mismatch/N2 antecedent-pronoun match across Mod and Comp structures therefore simply could not yield Mod- vs. Comp-modulated ERPDs in early and late anaphoric matches until *wh*-filler re-representations at gap sites enable the relevant processes. The processing of subject positions alone cannot carry these ERPDs induced by the timing of referential chain objects for Comps in syntactic binding supported by *wh*-filler computations. Indeed, no Mod- vs. Comp-modulated N1 antecedent-pronoun match vs. N1 antecedent-pronoun mismatch/N2 antecedent-pronoun match ERPD effects arose in the γ range at the matrix-clause and embedded-clause subjects.

Following Dekydtspotter et al. [27], we examined evoked γ power that reflects both time-locked and phase-locked oscillatory responses. We first computed the ERPs of each condition for each subject. Then, we extracted power information by convolving a family of Morlet wavelets of 7 cycles in .5 Hz steps within the selected time window of each EEG trial, which yielded the time-frequency information of the neural activity. The length of the wavelets was set as 3 standard deviations of the Gaussian kernel. At 60 Hz, the wavelet duration was 0.037 seconds. The spectral bandwidth was 17.143 Hz. We log-transformed (10*log10) the derived power in Fieldtrip to standardize the unit as decibels at each of the frequencies between 20 Hz and 100 Hz for each condition of each subject. The statistical analysis time window lasted 500ms starting at 50ms before the presentation of each verb, as we expect information prior to 200ms into a word to reflect predictive processing and information starting around 200ms into a word to reflect computations dependent on lexical information for the specific word [96]. These time windows capture processing moments where the bridge verb enables an embedded-clause dependency through an intermediate gap position, and when the embedded-clause verb enables a thematic gap position enabling the thematic integration of the *wh*-filler.

### Statistical analysis

We calculated Mod vs. Comp power differences between the N1 antecedent-pronoun match [(2a) – (2b)] and N1 antecedent-pronoun mismatch/N2 antecedent-pronoun match [(2c) – (2d)] conditions to address distinct allocations of resources in discourse-based vs. syntax-based anaphoric processes for Mods vs. Comps as in Table 3, reflecting different binding domains for prepositional Comps vs. clausal Mods. We then examined differences between Mod and Comp structures in N1 antecedent-pronoun match vs. N1 antecedent-pronoun mismatch/N2 antecedent-pronoun match. Data were analyzed with cluster-based nonparametric permutation tests to avoid the multiple comparison problem for our medium-density electrodes, on the assumption that the spatially adjacent channels exhibit similar spectral-temporal features [97–99]. We performed cluster-based permutation tests using Monte Carlo simulations with 10,000 random samplings for each channel-frequency-time triplet [99]. A reviewer wondered why we did not use an ANOVA given a 2x2 design and instead used paired-samples *t-*tests examining differences of differences. We follow the guidance of FieldTrip (https://www.fieldtriptoolbox.org/faq/stats/clusterstats_teststatistic/). As pointed out on the FieldTrip website, for a two-sample setting, *t* and *F* distributions are “interchangeable,” since *F *= *t*^2^. However, in cluster-based permutations, *t* and *F* value sums (cluster mass) for each iteration provide radically different null distributions, so that only the *t* version of the cluster-based permutation test “maintain[s] the statistical properties of the test (in terms of power).” Cluster-based permutation testing based on *F* values, therefore, is not recommended. Finally, we note that permutation tests provide the time window and electrodes within which a significant effect arises. They, however, lack precision as to the exact timing and location of effects [100]. Our discussion of the timing of ERPD effects is therefore limited to the window in which the effects are found.

At each critical segment (intermediate non-thematic and thematic gap positions), we conducted paired-samples *t-*tests within two bins (low γ 30–50 Hz and high γ 50–90 Hz). The two bins reflect frequency bands that have crucially been shown to be engaged in the processing of pronouns, with low γ frequencies first implicated in information retrieval followed by high γ frequencies in integration [22,29]. Non-thematic intermediate clause-edge gaps and thematic gaps reflect distinct theoretically dependent steps in filler-gap dependencies, with an intermediate non-thematic clause-edge gap dependent on an internal Merge approach, not required for effects obtained in thematic integration alone. The different positions therefore reflect different hypotheses about the nature of language computations [89]. The non-thematic intermediate gap position enabling the computation of a binding domain for the embedded clause is predicted to induce low-γ ERPDs in the retrieval of referential elements into an γ-implemented operational workspace inducing high-γ ERPDs as referential chain objects are generated. In contrast, the thematic gap position is predicted to induce high-γ ERPDs in the finalization of anaphora resolution. In view of the specificity of these positions associated with different processes supporting different research questions, motivated by a range of psycholinguistic research findings supporting a parse implicating the embedded clause edge [27,35–38,64,101] together with specific roles for low and high γ, we adopted a conservative Bonferroni correction of α = .025 for two frequency bands as most appropriate. A lower threshold of α = .0125 despite specific predictions for the two segments could mean that strongly suggestive ERPD effects with clear gap-position timing in the retrieval of referential elements in low γ and the computation of referential chain objects within high γ might be artificially dismissed, with their potential theoretical significance wrongly ignored.

## Results

**Fig 2:**
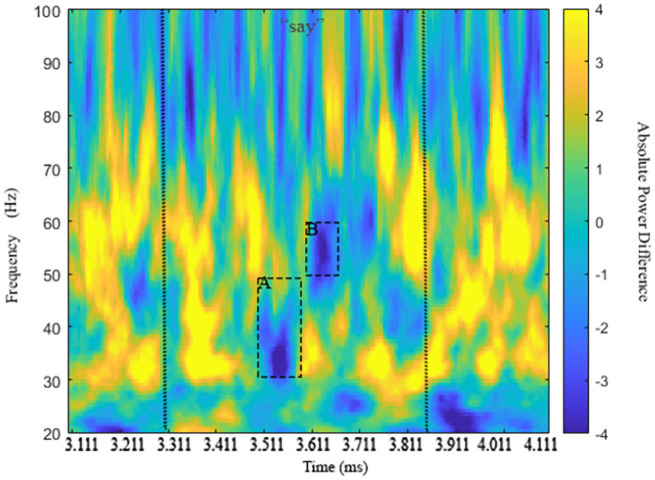
Time-frequency plot of Effects A (30-50 Hz) and B (50-60 Hz). Vertical lines indicate presentation of verb *say* (3307-3857ms).

**Fig 3:**
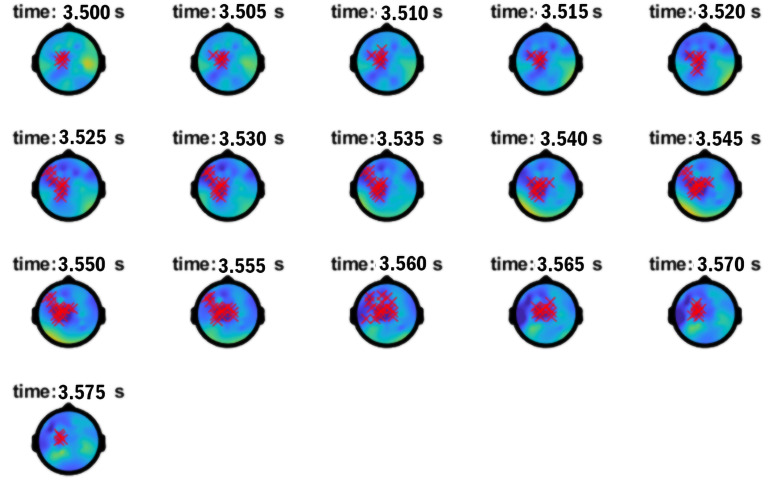
Topographical distribution of Effect A in low γ (30-50 Hz) at *say.*

**Fig 4:**
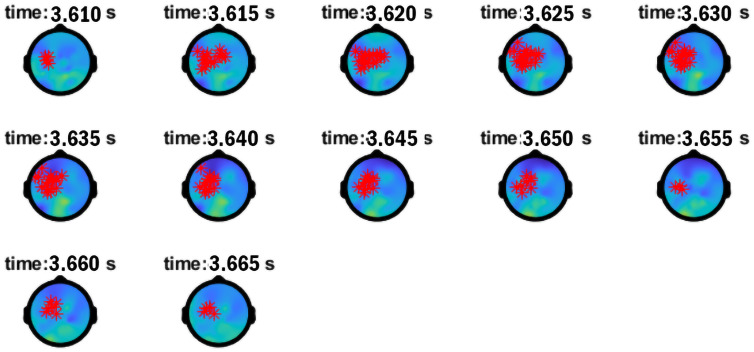
Topographical distribution of Effect B in high γ (50-60 Hz) at *say.*

As shown in Table 4, a significant ERPD effect showing the Mod vs. Comp mediation of N1 antecedent-pronoun match vs. N1 antecedent-pronoun mismatch/N2 antecedent-pronoun match in anaphora processing arose in low γ (within 30–50 Hz) lasting for 75ms between 3500–3575ms, 193ms into the verb *say* (presented 3307–3857ms). Fig 2 presents the time-frequency plot showing the low-γ Effect A. The low-γ Effect A occurred when the lexical information for the verb *say* was being retrieved, enabling access to clausal categories, required for the re-representation of a *wh*-filler into an embedded clause. Effect A exhibited a left centro-parietal scalp distribution (Fig 3). Within the 50–90 Hz bin, visual inspection of the time-frequency plot in Fig 2 made it clear that the low-γ Effect A was followed by a high-γ effect around 50–60 Hz. As reported in Table 4, a high-γ range 50–60 Hz Effect B started about 303ms into the bridge verb presentation lasting for 55ms between 3610–3665ms. Effect B exhibited a left frontal scalp distribution (Fig 4). This high-γ range Effect B matched the power distribution of Effect A, tied to higher power for Comps vs. Mods in N1 antecedent-pronoun match and lesser power for Comps vs. Mods in N1 antecedent-pronoun mismatch. Low-γ power ERPDs (Effect A) followed by similar high-γ-range power ERPDs (Effect B) also echo previously established patterns in anaphora resolution. Since anaphoric relations have been tied to increased power in low γ (around 40 Hz) for retrieval and in high γ (60–80 Hz) for integration [22], these effects tied to the modulation of anaphoric dependency by Mod vs. Comp structures are consistent with the computation of a referential chain object induced by the processing of *wh*-filler-gap dependencies through the clause edge in top-down left-corner parsing.

**Table 4:** Low-γ ERPD Effects A, B at the bridge verb and high-γ ERPD Effect C at the embedded verb.

Effect	Hz	*p*	*t*	SD	Timing ms (Duration)	Electrodes^a^ (Distribution)	Power differences (Mod – Comp)
Effect A	30-50	0.013	−480.07	0.0012	3500 - 3575 (75)	7 15 16 17 18 19 20 21 22 25 26 (left centro-parietal)	[(1a) - (1b)] = −2.3581 [(1c) - (1d)] = 1.7666
Effect B	50-60	0.0082	−375.23	0.0010	3610 - 3665 (55)	4 6 8 10 11 12 13 14 15 19 20 (left frontal)	[(1a) - (1b)] = −2.1004 [(1c) - (1d)] = 3.6714
Effect C	50-90	0.011	−414.64	0.0011	5000 - 5070 (70)	4 9 43 44 45 46 (left frontal/right temporo-parietal)	[(1a) - (1b)] = −2.1324 [(1c) - (1d)] = 1.3676

*Note.* α = .025. Verb *say* was presented 3307–3857ms. Embedded verb was presented 4960–5510ms.

^a^Electrodes reported are those that were most consistently activated within the time window of the effect.

**Fig 5:**
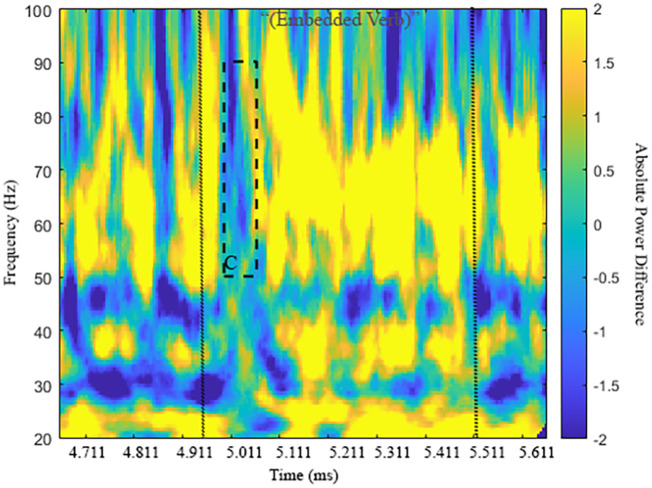
Time-frequency plot of Effect C in high γ (50-90 Hz). Vertical lines indicate presentation of embedded verb (4960-5510ms).

**Fig 6:**
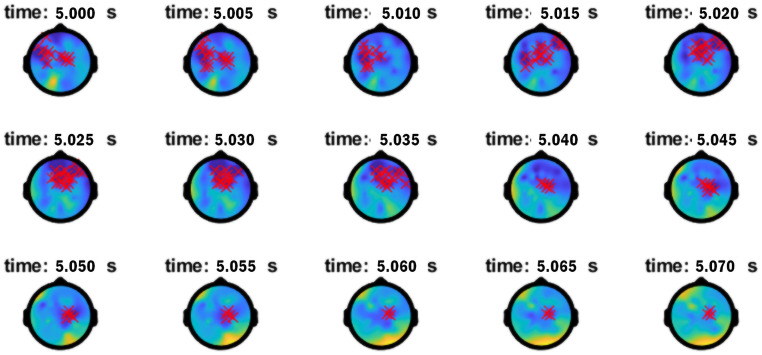
Topographical distribution of Effect C in high γ (50-90 Hz) at embedded verb.

Finally, these ERPD effects as an embedded-clause dependency was established were later echoed 40ms into the embedded-clause verb (presented 4960–5510ms). ERPD Effect C between 5000–5070ms was found 50–90 Hz for 70ms. The power distribution leading to this effect shown in Table 4 (row 4) fully echoes those already observed at the bridge verb *say*, again with greater power for Comps vs. Mods in N1 antecedent-pronoun match and greater power for Mods vs. Comps in N1 antecedent-pronoun mismatch/N2 antecedent-pronoun match. Fig 5 highlights the timing of this effect and activity across frequencies. Fig 6, however, highlights a left frontal and right temporo-parietal distribution as the anaphoric computations for Mods vs. Comps are finalized when the referential chain object needs to be integrated with the thematic processing of the *wh*-filler in the generation of a sentential interpretation.

All in all, Mod- vs. Comp-modulated anaphora-linked ERPD effects arose as the bridge verb induced an embedded-clause *wh*-filler-gap dependency and as a thematic object position was anticipated in early presentation of the embedded-clause verb segment. The power asymmetries that they represented were aligned with theoretical expectations for the computation of Comp-based referential binding relations dependent on *wh*-filler-gap dependencies vs. Mod-based discourse coreference relations. A low-γ ERPD effect at the beginning of a new computational cycle for the embedded clause is consistent with the low-γ retrieval of referential elements from working memory. The subsequent ERPD Effect B (50–60 Hz) at the bridge verb as an embedded clause *wh*-dependency is established enabling a Comp-based referential chain object combination in syntactic binding, together with a high-γ-range ERPD Effect C (50–90 Hz) as an embedded thematic verb is anticipated, are consistent with the role of high-γ activity in the construction of referential chain objects for binding and coreference.

## Discussion

This series of anaphora-linked Mod- vs. Comp-modulated ERPD effects echoed across γ frequencies began with an effect at 30–50 Hz in low γ as the bridge verb was accessed from the lexicon, leading to a matching ERPD Effect B at 50–60 Hz within the high-γ range. Then, a second high-γ ERPD effect at 50–90 Hz was also captured in anticipation of a verb phrase in defining a thematic gap position. The timing of these Mod- vs. Comp-modulated anaphora-linked ERPD effects highlights the role of γ oscillations in the implementation of referential relations dependent on the computation of *wh*-filler-gap dependencies. The high-γ ERPD effect at 50–90 Hz suggests the role of γ oscillations in the completion of a referential chain object in anaphora resolution as the processing of the embedded-clause verb phrase terminates the *wh*-filler-gap dependency. The timing of Mod- vs. Comp-modulated anaphora-linked ERPDs at gap sites seems highly suggestive of the reliability of these ERPDs in highlighting the γ-implementation of basic operations to the generation of referential objects in the computation of filler-gap dependency resolution. Crucially, pronouns need to be contextually identified in syntactic binding or discourse coreference subject to structural constraints. Hence, Mod- vs. Comp-modulated anaphora-linked γ ERPDs timed with gap-site processing in *wh*-filler re-representation highlight a Comp-modulated syntactically guided interpretation of pronouns computed within a γ-implemented operational workspace enabling discourse-based vs. syntax-based referential processes for Mods vs. Comps as part of a processing loop architecture. These Mod- vs. Comp-modulated anaphora-linked ERPD effects contribute a second set of neurocognitive evidence to psycholinguistic processing data addressing *wh*-filler re-representations in intermediate and thematic gaps in the processing of bi-clausal filler-gap dependencies. These theoretically aligned Mod- vs. Comp-modulated anaphora-linked ERPDs across time and frequency are consistent with the hypothesis that γ oscillations implement basic language operations as in Murphy’s ROSE model [13], showing the role of such a design in the parsing of referential relations in complex dependencies. The timing and nature of these ERPDs clearly align with psycholinguistic research. These Mod- vs. Comp-modulated anaphora-linked γ ERPD effects emerged as referential chain objects in Comp-enabled syntactic binding with specific structurally determined timing vs. Mod-enabled discourse coreference. Thus, grammatical requirements on pronouns and *wh*-filler structures, together with general requirements on variables interpreted in discourse or syntax, induced these effects. The power asymmetries across intermediate and thematic gap Mod- vs. Comp-modulated anaphora-linked ERPD effects are aligned with the role of low-γ and high-γ oscillations in the computational steps establishing anaphoric relations in grammar vs. discourse. Across low-γ and high-γ frequencies, these ERPDs involved greater resources devoted to matrix-clause antecedent anaphora in Comps (relative to Mods) and to embedded-clause antecedent anaphora in Mods (relative to Comps). Mod vs. Comp asymmetries involved greater power for the syntactically determined creation of referential grammatical objects for Comps (2b) vs. a pre-established coreference in Mods (2a). They also involved greater power for the active processing of the discourse referent for the pronoun in later identification (2c) vs. a pre-established binding chain object (2d), since the latter implicates reduced resources within the embedded clause compared with the coactivation of multiple discourse referents in coreference.

Hence, the asymmetries in these ERPDs point to the role of a cortical γ-implemented operational workspace in the creation of referential chain combinations for grammar vs. discourse and the referential value assignment to the combination necessitated in final integration. The nature of the power asymmetries in these effects echoed the nature of the power asymmetries in the Mod- vs. Comp-modulated anaphora-linked γ ERPDs documented in Dekydtspotter et al. [27] for French, although those Mod- vs. Comp-modulated anaphora-linked ERPDs arose at the subordinator. The English tense marker *did* in interrogative position unambiguously requires a bare root thematic verb like *say.* In contrast, the French tense marker *a* ‘has’ is ambiguous as a present tense main verb or past tense auxiliary. This difference could impact the speed of information flow for verbal elements in the two languages. Despite this difference in timing, the consistent alignment of γ ERPD effects with computations grounded in psycholinguistics across stimuli and distinct populations suggests the role of γ oscillations across syntax and semantics, including referential processes.

Anaphora-linked γ power differences have indeed been associated with bottom-up referential processing of pronouns. Nieuwland and Martin [22] discussed ERPDs in items such as *The boy thought that he/she would win the race*, in which *he* within a Comp clausal structure allows for an automatic binding computation in syntactic anaphora, whereas *she* requires the accommodation of a contextual referent for the pronoun in deixis. Nieuwland and Martin reported that “Beamformer analysis in high-density EEG localized the increase around 40 Hz to left posterior parietal cortex and the increase in the 60–80 Hz range to left inferior frontotemporal cortex” [22, p. 904]. The distribution of the effects found here, therefore, also lines up with regions tied to referential processing in general. Using fMRI, Nieuwland et al. [102] reported that referentially unambiguous pronouns in anaphora produced more solid BOLD responses than did ambiguous pronouns in left and right inferior frontal brain regions and in the anterior temporal lobe. The distribution of ERPD effects reported here at the clause edge in left centro-parietal electrodes (Effect A) and left frontal electrodes (Effect B), together with left frontal and right temporo-parietal electrodes (Effect C) at the embedded-clause verb, therefore, is not only tied to re-representations in *wh*-dependency processing for the embedded clause but also consistent with documented sites engaged in anaphora processing.

While increased γ power has also been associated with the tracking of semantic fitness in bottom-up processing, in our experimental design the Mod vs. Comp expressions provide the same information in structurally alternative ways. Semantic fitness, therefore, cannot account for the ERPD effects reported here; they instead point to alternative computational routes taken as a function of lexico-grammatical specifications. Mod vs. Comp modulations of anaphora-linked ERPDs in γ, in the absence of any differences in the synchronous bottom-up processing of phrasal arrays between Mod and Comp conditions, suggest the role of γ oscillations in the creation and integration of referential chain objects implementing referential relations in the ongoing processing of the sentence [6,13]. These effects linked to *wh*-filler contents at gap sites are also inconsistent with approaches to language computations that claim no re-representation of *wh*-fillers [89]. Addressing the nature of γ computations, Murphy [13] argued that low-γ activity participates in cross-rhythm interactions (see also [103]). In contrast, high/broadband γ implements operations, with spiking rate signaling the activation of stored representations in the transfer of elementary units into a computational workspace: “The integration between broadband and slow (30-50 Hz) γ is an interesting case in this respect. Broadband γ correlates with neuronal spiking; spiking is phase-locked to slow γ; and, in turn, fast γ exhibits coupling with slow γ” [13, p. 10]. Low-γ 30–50 Hz ERPDs followed by 50–60 Hz ERPDs within the high γ range distributed in centro-parietal electrodes at the edge of an embedded clause are consistent with the return of grammatical syn-sem expressions from working memory in low γ and their combining within high γ. High-γ (50–90 Hz) ERPDs largely found in right-hemisphere (temporo-parietal) electrodes at the embedded thematic verb suggest the role of γ oscillations in the final computational steps with the assignment of discourse referents or entities in a cognitive model for a possible world in the resolution of syntax vs. discourse anaphora in contextual-semantic evaluation of the sentence.

In Murphy’s [13] oscillatory model, a γ-implemented cortical operational workspace in interaction with structural memory within a cortical-subcortical loop enables recursion together with the nesting of relations in complex dependencies [6,28]. We considered how a γ-implemented cortical operational workspace allows basic hierarchical operations in the creation of syn-sem objects and how it enables a left-corner parsing system consistent with a Minimalist design as in Tables 1 and 2. We next considered how a γ operational workspace enables relations (Table 3). In syntactic binding, the combination {Frank, him} of syn-sem referential elements for the expressions *Frank* and *him* must receive a referent x for an entity named *Frank* in sequences of coactivation, yielding a referential chain object {x, {Frank, him}} as an anaphoric relation is computed in sentential interpretation. In the γ-implemented cortical operational workspace, coactivations of various cortical nodes enable various syn-sem objects to be normalized to basic elements that can therefore be combined into referential chain objects and assigned a referential value. Likewise, the identification (x = y) of the discourse referents for *Frank* and *him*, respectively, in discourse coreference can also be achieved by the same type of basic cortical γ computations. Coreference, therefore, involves mapping a combination of discourse referents {x: Frank, y: him} to an entity (f) in a cognitive model for a possible world with someone named Frank [55] (Table 3). This referential chain object {f, {x: Frank, y: him}} can then be integrated into working memory to enable the consolidation of a discourse representation structure in interpretation. Such anaphoric relations in binding and coreference must therefore involve a Markovian hierarchical processing system compatible with computational Bayesian inference approaches to information processing by cortical circuitry [88]. As Murphy [104] and Lasnik and Uriagereka [105] point out across distinct language sciences, the syn-sem symbolic system for language enabling hierarchical tree structures and relations is fully reconcilable with statistically based information processing mechanisms.

## Conclusion

Fulfilling our aims, we first empirically documented Mod- vs. Comp-modulated γ-range activity in referential computations with its timing consistent with top-down structure building enabling anaphora processing specifically linked to *wh*-filler re-representations at the clause-edge intermediate gap and in anticipatory thematic gap processing. Second, we conceptually characterized how implementational cortical circuitry may implement referential chain objects enabling anaphoric relations in syntax and discourse. The γ ERPDs found here suggest that γ oscillations play a role in the creation of basic relational objects with discourse and syntactic elements as *wh*-fillers are re-represented. They are consistent with γ effects in the interpretation of pronouns with narrowband, low-γ effects in reinstatement from working memory and high/broadband-γ effects tied to semantic integration. γ oscillations in the top-down processing of referential chain objects in anaphoric relations supports the claim of a γ-implemented operational workspace in language generation and interpretation [6,13,24,27]. Third, the Mod vs. Comp modulation of anaphora-linked ERPD effects in γ reported here for English echo those of Dekydtspotter et al. [27] for French, while the slightly different timing seems consistent with different presentation of information by language-specific vocabulary items.

The integration of neuroscience and linguistics still presents a significant hurdle [106], as is evident in the current debate on the nature of γ oscillations. Such a complex mapping can only be slowly advanced. As Matchin and Hickok noted, “the detail of specific operations...are instantiated by subtler biophysical properties... such as network connectivity patterns or cortical oscillations” [107, p. 1486]. Despite its limitations, noninvasive EEG can nevertheless inform a highly specific hypothesis about language computations, thereby addressing aspects of the nature of language in brain activity that can thereafter be investigated in greater detail in other more intrusive ways. Indeed, Mod- vs. Comp-modulated anaphora-linked γ ERPD effects with timings reflecting nested referential dependencies in *wh*-movement certainly support the “idea of implementing recursion through a two-level abstract chunking structure and a backward loop” [108, p. 724]. The role of γ oscillations in the nesting of referential relations within complex dependencies suggests a γ-implemented computational cortical workspace for basic operations as per Murphy’s ROSE model in which high/broadband-γ oscillations implement object construction, with low-γ sites tied to low rhythms in working memory for items and structures. A γ-implemented operational workspace within a loop architecture for language seems indeed central to an explanation of the expressive power of human language.
